# In Vitro Cultivation of Limbal Epithelial Stem Cells on Surface-Modified Crosslinked Collagen Scaffolds

**DOI:** 10.1155/2019/7867613

**Published:** 2019-04-01

**Authors:** Michel Haagdorens, Vytautas Cėpla, Eline Melsbach, Laura Koivusalo, Heli Skottman, May Griffith, Ramūnas Valiokas, Nadia Zakaria, Isabel Pintelon, Marie-José Tassignon

**Affiliations:** ^1^Faculty of Medicine and Health Sciences, Department of Ophthalmology, Visual Optics and Visual Rehabilitation, University of Antwerp, Campus Drie Eiken, T building, T4-Ophthalmology, Universiteitsplein 1, 2610 Antwerp, Belgium; ^2^Department of Ophthalmology, Antwerp University Hospital, Wilrijkstraat 10, 2650 Antwerp, Belgium; ^3^Department of Nanoengineering, Center for Physical Sciences and Technology, Savanorių 231, 02300 Vilnius, Lithuania; ^4^Ferentis UAB, Savanorių 235, 02300 Vilnius, Lithuania; ^5^Center for Cell Therapy and Regenerative Medicine, Antwerp University Hospital, CCRG-Oogheelkunde, Wilrijkstraat 10, 2650 Edegem, Belgium; ^6^Faculty of Medicine and Health Technology, Tampere University, Arvo Ylpön katu 34, 33014, Finland; ^7^Maisonneuve-Rosemont Hospital Research Centre and Department of Ophthalmology, University of Montreal, Montreal, QC, Canada H1T 4B3; ^8^Laboratory of Cell Biology and Histology, Antwerp University, Campus Drie Eiken, T building, T1-Veterinary Sciences, Universiteitsplein 1, 2610 Antwerp, Belgium

## Abstract

**Purpose:**

To investigate the efficacy of recombinant human collagen type I (RHC I) and collagen-like peptide (CLP) hydrogels as alternative carrier substrates for the cultivation of limbal epithelial stem cells (LESC) under xeno-free culture conditions.

**Methods:**

Human LESC were cultivated on seven different collagen-derived hydrogels: (1) unmodified RHC I, (2) fibronectin-patterned RHC I, (3) carbodiimide-crosslinked CLP (CLP-12 EDC), (4) DMTMM- (4-(4,6-dimethoxy-1,3,5-triazin-2-yl)-4-methyl-morpholinium-) crosslinked CLP (CLP-12), (5) fibronectin-patterned CLP-12, (6) “3D limbal niche-mimicking” CLP-12, and (7) DMTMM-crosslinked CLP made from higher CLP concentration solution. Cell proliferation, cell morphology, and expression of LESC markers were analyzed. All data were compared to cultures on human amniotic membrane (HAM).

**Results:**

Human LESC were successfully cultivated on six out of seven hydrogel formulations, with primary cell cultures on CLP-12 EDC being deemed unsuccessful since the area of outgrowth did not meet quality standards (i.e., inconsistence in outgrowth and confluence) after 14 days of culture. Upon confluence, primary LESC showed high expression of the stem cell marker ΔNp63, proliferation marker cytokeratin (KRT) 14, adhesion markers integrin-*β*4 and E-cadherin, and LESC-specific extracellular matrix proteins laminin-*α*1, and collagen type IV. Cells showed low expression of differentiation markers KRT3 and desmoglein 3 (DSG3). Significantly higher gene expression of KRT3 was observed for cells cultured on CLP hydrogels compared to RHC I and HAM. Surface patterning of hydrogels influenced the pattern of proliferation but had no significant effect on the phenotype or genotype of cultures. Overall, the performance of RHC I and DMTMM-crosslinked CLP hydrogels was equivalent to that of HAM.

**Conclusion:**

RHC I and DMTMM-crosslinked CLP hydrogels, irrespective of surface modification, support successful cultivation of primary human LESC using a xeno-free cultivation protocol. The regenerated epithelium maintained similar characteristics to HAM-based cultures.

## 1. Introduction

Located at the corneoscleral limbus, limbal epithelial stem cells (LESC) play a pivotal role in rejuvenating the corneal epithelium and keeping the cornea healthy, transparent, and avascular [1, 2]. Damage to the LESC or their stem cell niche may lead to limbal stem cell deficiency (LSCD). This condition is characterized by conjunctivalization and cicatrization of the cornea and may result in reduced vision, pain, and photophobia [3]. Historically, surgical treatment of patients suffering from LSCD includes conjunctival limbal grafting or keratolimbal allografting.

Since its introduction in 1997 [4], cultivated limbal epithelial transplantation (CLET) has shown to be an effective therapy for LSCD, with clinical trials reporting an average success rate of 70% [5]. In CLET, a small limbal biopsy is cultivated ex vivo on a stem cell carrier, after which cultured cells are grafted into the patient's diseased eye. The stem cell carrier most frequently used in these trials is the human amniotic membrane (HAM). Harvested by caesarian section, HAM has been used for many years in ocular surgery [6, 7]. It has the advantage of having anti-inflammatory, antimicrobial, and antiangiogenic properties [7, 8]. However, being a biological membrane, procurement of HAM requires costly donor screening for potential infectious pathogens [9]. In addition, standardization of HAM procedures is difficult due to inter- and intradonor variabilities in membrane thickness, mechanical properties, optical characteristics, and growth factor release [10–13]. Furthermore, *in vitro* processing remains labor intensive, costly, and challenging [9]. These limitations hamper the application of HAM in ocular tissue engineering. Other stem cell carriers such as fibrin and siloxane hydrogel contact lenses have been used in human clinical trials [5]. In 2015, Holoclar® (Chiesi, Italy), a technique in which limbal cells are expanded on fibrin scaffolds, was conditionally approved for release in Europe as the first commercially available stem cell therapy for LSCD. Nevertheless, use of this medicinal product is restricted to autologous stem cell transplantation in unilateral cases after chemical or thermal burn. Furthermore, fibrin hydrogels require the application of xenogenic culture protocols that involve murine 3T3 feeder layers, which brings into question safety of the end-product. Therefore, a safe and standardized therapy that targets all LSCD patients has yet to be developed.

Various biomaterials have been proposed as alternative carriers to the use of HAM and fibrin in corneal tissue engineering [5, 14]. A promising approach is the application of collagen hydrogels, as these are characterized by inherent biocompatibility and cost effectiveness [15, 16]. In 2009, the group of Fagerholm et al. were the first to report the successful implantation of acellular recombinant human collagen type III (RHC III) hydrogels, crosslinked by 1-ethyl-3-(3-dimethyl aminopropyl) carbodiimide/N-hydroxysuccinimide (EDC/NHS), as corneal stromal substitutes in humans [17]. In subsequent reports, RHC III-based hydrogels were implanted in 20 patients, with collagen being sourced from yeast in each of these cases [18–20]. After surgery, implants supported full epithelial regeneration, though slow reepithelialization rates could be noted, with full epithelial regeneration taking up to one year [20]. Additional exploration of RHC III-based hydrogels showed that surface modification, by means of fibronectin microcontact printing (F-*μ*CP), improved reepithelialization rates *in vitro* [21]. Even though F-*μ*CP of RHC III implants has yet to be validated *in vivo*, these results indicate the potential of surface modification in collagen-based corneal regeneration.

In recent years, alternative collagen sources have shown great promise in tissue engineering, including fully synthetic collagen-like peptide (CLP) and plant-derived RHC type I (RHC I) [22–25]. CLP [26] was introduced as a shorter and fully customizable alternative to RHC III peptide. As a synthetic peptide, CLP makes room for ready and scaled-up production. When tested as a corneal construct in an animal model, CLP proved to be functionally equivalent to RHC III, except for mechanical strength for which CLP underperformed [27, 28]. These reports provide a proof-of-principle and indicate that it is worthwhile exploring the versatility of CLP hydrogels as a scaffold for LESC cultivation.

Another type of collagen that recently became available is tobacco plant-derived RHC I [25]. Even though plant-derived RHC I has shown promise in experimental skin engineering and drug delivery [22, 29, 30], its application in ocular tissue engineering remains to be validated. Previous research compared the *in vitro* and *in vivo* performance of yeast-extracted RHC I and RHC III corneal constructs and concluded that both materials perform fairly similarly, though RHC III displayed marginally superior mechanical properties [31, 32]. These results, in combination with collagen type I being the most abundant protein of the native corneal stroma [33], suggest that plant-derived RHC I might offer greater potential in ocular tissue engineering. Our previous research demonstrated that plant-derived RHC I hydrogels are mechanically stable, transparent, and nongenotoxic and show good biocompatibility *in vitro* and *in vivo*. Even though plant-derived RHC I and CLP hydrogels appear promising substrates, both materials remain to be validated as carrier membranes for LESC cultivation.

Therefore, the aim of this study was to investigate *in vitro* performance of 4-(4,6-dimethoxy-1,3,5-triazin-2-yl)-4-methyl-morpholinium chloride- (DMTMM-) crosslinked CLP hydrogels, EDC/NHS-crosslinked CLP hydrogels, and EDC/NHS-crosslinked plant-derived RHC I hydrogels with regard to immortalized human corneal epithelial cell (iHCEC) and primary human limbal epithelial cell cultivation. The effect of surface topography and patterning was investigated for both hydrogels. All data were compared to HAM, the current gold standard in CLET.

## 2. Materials and Methods

The study followed the tenets of the Declaration of Helsinki and was approved by the Antwerp University HospitalEthical Committee (EC: 14/30/319).

### 2.1. Materials

Plant-derived RHC I and PEGylated CLP were provided by Collplant (Ness Ziona, Israel) and Ferentis (Vilnius, Lithuania), respectively. Laboratory plastic was purchased from VWR (Radnor, PA, USA), Greiner Bio-One (Kremsmünster, Austria), or PerkinElmer (Waltham, MA, USA). Unless stated otherwise, all inorganic salts, enzymes, basic chemicals, Triton X, 4′,6-diamidino-2-fenylindool (DAPI), N-hydroxysuccinimide (NHS), N-(3-dimethylaminopropyl)-N′-ethylcarbodiimide hydrochloride (EDC), 4-(4,6-dimethoxy-1,3,5-triazin-2-yl)-4-methylmorpholinium chloride (DMTMM), and CellCrown inserts were purchased from Sigma-Aldrich (St. Louis, MO, USA). Materials obtained from Thermo Fisher Scientific (Waltham) include phosphate-buffered saline (PBS), PrestoBlue, Dulbecco's modified Eagle's medium (DMEM), keratinocyte serum-free medium, Live/Dead staining kit, Alexa Fluor® 568 hydrazide sodium salt, antibiotics, glycerol, and UltraPure distilled water (DW). Optimum cutting temperature (OCT) formulation was purchased from Sakura Finetek Europe (Zoeterwoude, the Netherlands); nitrocellulose paper and filter sterilizers were from Merck Millipore (Darmstadt, Germany); polydimethylsiloxane (PDMS) was from Dow Corning (Midland, MI, USA); balanced salt solution (BSS) was from Alcon (Fort Worth, TX, USA); CnT-prime medium (CnT-PR) was from CELLnTEC (Bern, Switzerland); PBS/glycerol Citifluor was from Citifluor Ltd. (London, UK); and RNeasy Mini Kit was from QIAGEN (Hilden, Germany). Human blood fibronectin was obtained through YO Proteins AB (Huddinge, Sweden) whereas bovine fibronectin was delivered by Cytoskeleton Inc. (Denver, CO, USA). iScript™ Advanced cDNA Synthesis kit, SsoAdvanced™ Universal SYBR® Green Supermix, and oligonucleotide primers were obtained from Bio-Rad (Hercules, CA, USA), unless stated otherwise. ΔNp63*α* primer was purchased from Eurogentec (Liege, Belgium) (Table 1). Antibodies used for immunohistochemistry and its dilutions are listed in supplementary Table S1.

### 2.2. Cell Carrier Preparation

#### 2.2.1. Human Amniotic Membrane

With ethical approval from the UZA ethical committee (EC: EC: 14/30/319) and signed written informed consent from donors, amniotic membranes were obtained from women undergoing scheduled caesarean sections. HAM was cryopreserved and processed using previously described methods [34]. In brief, the HAM was peeled away from the chorion and washed in BSS containing penicillin/streptomycin and amphotericin B. It was then flattened onto a sterilized nitrocellulose filter paper and cryopreserved at −80°C in 1 : 1 solution containing DMEM and glycerol. The HAM was thawed 48 hrs before use and washed three times in saline, after which it was treated with 50 mL Thermolysin solution (0.12 mg/mL) for 8 min to remove amniotic epithelium. After enzymatic digestion, the membrane was washed in 0.01 M PBS after which orientation of the membrane was tested with the previously described 'cotton swab technique' [34]. When the de-epetheliazed surface was identified to be superior, the membrane was fixed in an interlockable ring [34]. Prior to primary cell cultivation, the HAM was immersed in the respective culture medium containing 5% human AB serum (hAB) for at least 24 hrs.

#### 2.2.2. Recombinant Human Collagen Hydrogels

Plant-derived RHC I was obtained as a solution in 10 mM HCl. A 3 : 7 solution of pure (100%) ethanol/collagen was stirred for 30 min at 25°C, after which fibrillogenesis buffer (160 mM Na_2_HPO_4_ and 100 mM NaOH at pH 7.5) was added at a ratio of 1 : 10 *v*/*v* to the original collagen-HCl volume and stirred for 2 more hours. Water-diluted EDC and NHS were added for a final concentration of 50 mM EDC and 100 mM NHS and stirred for 24 hrs at 4°C. All stirring was performed using a magnetic stirrer at 200 rpm. After 24 hrs, excess EDC/NHS was washed out with DW in 6 cycles. One cycle consists of centrifugation at full speed (10 min, 5.000 rpm), discarding the supernatant and resuspending the collagen in 40 mL DW. At cycle 6, the collagen suspension was transferred to a Teflon mold and left air drying under a sterile hood. When fully dried, collagen gels were collected and stored in 100% ethanol until further use. Rehydration of gels was performed by 5 individual washes in PBS, each lasting 2 hrs. For cell cultivation, the hydrogels were soaked thrice for 2 hrs in the respective culture medium and immobilized with a CellCrown or interlockable ring.

#### 2.2.3. Collagen-Like Peptide Hydrogels

CLP peptide synthesis, conjugation with PEG maleimide, and CLP-PEG hydrogel fabrication were described in the study of Islam et al. [21]. Briefly, 500 mg of aqueous solution of 18% or 12% (*w*/*w*) CLP-PEG was dispensed in a 2 mL glass syringe, and either EDC/NHS or DMTMM was added to the syringe mixing system. The molar equivalents of CLP-PEG-NH_2_ : EDC were 1 : 2 and the molar ratio of EDC : NHS was 1 : 1. For CLP-PEG-NH_2_ : DMTMM, the molar ratio was 1 : 2. All reagents were thoroughly mixed prior to casting the hydrogel into thin flat sheets. Alternatively, the hydrogel was molded in a PDMS mold with a surface topography of 50 *μ*m wide and 20 *μ*m deep grooves. All CLP-PEG hydrogel sheets were cut into 15 mm diameter disks using a trephine and kept in a PBS buffer. Hydrogel sheet thickness and the groove topography were measured using an Olympus BX51 upright microscope equipped with a Peltier-cooled Fview II CCD camera (Olympus, Tokyo, Japan).

Prior to cell cultivation, CLP hydrogels were soaked in respective culture medium for 96 hrs (4 days). CLP hydrogels did not require fixation to allow cell cultivation.

#### 2.2.4. Surface Micropatterning

RCH I hydrogels were rehydrated in PBS and cut into approximately 20 × 25 mm^2^ sized pieces. Microcontact printing (*μ*CP) onto RHC I and CLP hydrogel surfaces was carried out as described previously [21]. Briefly, native surface carboxyl groups of RHC I and CLP-PEG hydrogels were activated by applying 10 mM EDC and 2.5 mM NHS in 0.1 M PBS (pH 5.7) for 15 min. The hydrogels were then washed with fresh PBS (pH 5.7). The surface of each sample was dried in a N_2_ stream, keeping the material bulk hydrated. Stamps were made of PDMS, with rectangular stamps (~16 × 23 mm) used for RHC I and 12 mm diameter disks for CLP-PEG printing. The stamps contained surface topography of protruding 30 *μ*m wide stripes with 60 *μ*m spaces in between. They were inked by applying 0.1 mg/mL human blood fibronectin solution for 5 min. For pattern visualization, ink contained 0.01 mg/mL of HiLyte488 dye-marked fibronectin. After inking, the PDMS stamp was brought into contact with the activated and dried hydrogel surface for 5 min. Subsequently, the remaining unreacted hydrogel surface was passivated by applying 10 mM PEG_3_NH_2_ (Molecular Biosciences, Boulder, CO, USA). The samples were washed with fresh PBS (pH 8.0) buffer and stored at 4°C until further use.

To investigate reproducibility of F-*μ*CP patterning, the patterned hydrogels were imaged using an Olympus BX51 upright microscope. Fluorescence images of fibronectin-HiLyte488 patterns were acquired and analyzed using the Stream Motion software (Olympus). Fibronectin pattern quality was assessed manually, using stitched fluorescence microscopy images taken over the entire printed hydrogel surface area. Then the actual printed surface area was calculated by subtracting any defects from the total area occupied by the surface topographic features on the PDMS stamp.

An overview of hydrogel composition and their respective abbreviation is provided in Table 2. HAM serves as a control. Hydrogels are (1) unmodified RHC I, (2) RHC I that was F-*μ*CP (RHC I F-*μ*CP), (3) EDC/NHS-crosslinked CLP (CLP-12 EDC), (4) DMTMM-crosslinked CLP (CLP-12), (5) CLP-12 with surface F-*μ*CP (CLP-12F-*μ*CP), (6) CLP-12 with 3D-grooved surface topography (CLP-12 3D), (7) and DMTMM-crosslinked CLP at a CLP stock concentration of 18% (CLP-18).

### 2.3. Cell Cultivation

#### 2.3.1. Immortalized Corneal Epithelial Cell Cultivation

For *in vitro* biocompatibility testing, immortalized human corneal epithelial cells (iHCECs) [35] were seeded onto the membranes and cultivated in keratinocyte serum-free medium. Cells were cultured in a humidified 37°C (5% CO_2_) incubator. To perform live cell imaging, green fluorescence protein- (GFP-) transduced iHCECs (GFP-iHCECs) [21] were cultured using the same cultivation protocol.

#### 2.3.2. Primary Limbal Epithelial Cell Cultivation

Cadaveric donor eyes were collected from the cornea tissue bank of the Antwerp University Hospital. The donor age ranged from 49 to 90 years with an average of 74 years. All donor eyes were processed within 32 hrs postmortem. In brief, the eyes were enucleated, transferred in 0.9% NaCl, and stored at 4°C. The eyes were disinfected for 1 min in povidone iodine 0.5%, after which they were rinsed 4 times in PBS. Biopsies of ≤2 mm^2^ were taken from the superior and inferior keratolimbal regions and washed 6 × 10 min in CnT-PR (CELLnTEC) at 4°C. Biopsies were then placed epithelial side down on the tested carrier materials (Table 2) and cultivated for 14 days at 37°C, 5% CO_2_, and 95% humidity. For cultivations on HAM and RHC I, 1% hAB was added to culture medium. Culture medium was changed every other day. The first 3 days, cells were cultivated at an air-liquid interface to allow biopsy attachment. Onwards, volume of the medium was increased to submerge cultures. At day 14 (or earlier if confluent), cells were characterized through immunohistochemistry and reverse transcriptase PCR (RT-PCR) analyses.

### 2.4. In Vitro Biocompatibility Testing

To assess *in vitro* biocompatibility of collagen-based hydrogels, samples of 6 mm diameter were punched out of the membranes and placed into 96-well plates. iHCECs were seeded onto the materials at a density of 5000 cells per membrane (*n* = 3) and cultured up to 4 days (96 hrs). Cell cultures on HAM served as a control. At 24 hrs, 48 hrs, 72 hrs, and 96 hrs of cultivation, a PrestoBlue cell metabolic activity assay was performed according to the manufacturer's protocol. In brief, PrestoBlue was added (1 : 10 *v*/*v*) to the cultures and incubated for 35 minutes. The supernatant was transferred to an opaque 96-well plate, and fluorescence was read at 590 nm with VICTOR^3^. Supplementary Live/Dead staining was performed at 48 hrs of cultivation, where cells were double stained with calcein acetoxymethyl (Calcein AM) and ethidium homodimer-1 (EthD-1). Cells cultured on tissue culture plastic (TCP) and treated with 0.1% saponin for 20 min at 37°C were used as positive controls for EthD-1. For PrestoBlue analysis, independent nonparametric *t*-testing was performed using the SPSS 24 Kruskal-Wallis test (IBM Corp., NY, USA) and Prism 5 (GraphPad Software, CA, USA). *p* < 0.05 was considered significant.

To evaluate the pattern of proliferation, live cell imaging of GFP-iHCECs [21] was performed. Hydrogel samples of 15 mm diameter were placed into 12-well plates. RHC I hydrogels were fixated with CellCrown inserts. GFP-iHCECs were seeded onto the materials at a density of 10.000 cells per membrane. Live cell imaging was performed in an incubator that was mounted on a confocal laser scanning microscope (Eclipse Ti microscope, Nikon, Tokyo, Japan; UltraVIEW VoX, PerkinElmer). The microscope recorded images at 90-minute intervals for 3 days (72 hrs). Images obtained at 72 hrs of culture were analyzed with ImageJ (National Institutes of Health, Bethesda, MD, USA) to calculate the percentage area of confluence. Of each culture condition, images of 6 different sites were analyzed. To detect statistical significance, independent nonparametric *t*-testing was performed using the Mann-Whitney *U* test in Prism 5 (GraphPad Software, CA, USA). Live cell imaging was not performed for HAM as the composite graft was not compatible with the microscope setup.

After 72 hrs of imaging, cells were kept in culture until day 4, when samples were fixed in 2.5% glutaraldehyde solution in 0.1 M sodium cacodylate buffer (pH 7.4), and processed for scanning electron microscopy (SEM) and transmission electron microscopy (TEM).

### 2.5. Electron Microscopy

#### 2.5.1. Scanning Electron Microscopy

For SEM, fixed samples were rinsed in 7.5% saccharose in 0.1 M cacodylate buffer, pH 7.4, and then dehydrated through an ascending ethanol gradient (50% ethanol 10 min; 70% - 90% - 95% ethanol 15 min each; 100% ethanol 3 × 30 min). After critical point drying, samples were mounted on a SEM grid and shutter coated with 20 nm gold. Images were recorded with a SEM 515 Microscope (Philips, Eindhoven, the Netherlands).

#### 2.5.2. Transmission Electron Microscopy

Samples were postfixed in 1% OsO_4_ solution and dehydrated in an ethanol gradient (50% - 70% - 90% - 95% ethanol for 15 min each, 100% ethanol for 4 × 20 min). Samples were embedded in EMbed 812 (Electron Microscopy Sciences, Hatfield, Pennsylvania), sectioned, and stained with lead citrate. Slides were examined using a Tecnai G2 Spirit BioTWIN Microscope (FEI, Eindhoven, the Netherlands) at 120 kV.

### 2.6. Characterization of Primary LESC Cultures

#### 2.6.1. Immunohistochemistry

For immunohistochemistry, cultures were fixed in 100% ethanol for 10 min at -20°C and rinsed thrice in PBS for 10 min each. Samples were embedded in OCT compound and stored at -80°C. Five cryostat sections (13 *μ*m thick) of each sample were mounted on poly-L-lysine-coated microscope slides, dried at 37°C for 2 hrs, and processed for fluorescence immunolabeling. Sections were then permeabilized with Triton X 1% for 25 min. Primary antibodies were incubated overnight at 4°C. Anti-ΔNp63, anti-cytokeratin 3 (KRT3), anti-laminin, anti-KRT14, anti-collagen type IV (Coll-IV), anti-integrin-*β*4 (INTB4), anti-desmoglein 3 (DSG3), and anti-E-cadherin (E-cad) served as primary antibodies (supplementary Table S1). Fluorescent secondary and tertiary antibody labeling was incubated for 2 hrs at 4°C. Nuclei were counterstained using DAPI, and sections were mounted with Citifluor. Images were recorded with confocal microscopy.

#### 2.6.2. RNA Extraction, Reverse Transcription, and Polymerase Chain Reaction

Prior to RNA extraction, cultures were rinsed once with 0.1 M PBS, preheated at 37°C. Cells were incubated with RNA lysis buffer, and total cell RNA was extracted, following RNeasy Mini Kit-enclosed guidelines. Total RNA was diluted in 14 *μ*L water, and purity was evaluated from the 260/280 ratio of absorbance (1.80–2.00) using the NanoDrop™ spectrophotometer (Thermo Fischer Scientific). cDNA was synthesized from 10 *μ*L of total RNA using iScript™ Advanced cDNA Synthesis kit and CFX96™ thermocycler (Bio-Rad), according to the manufacturer's protocol. cDNA was diluted to a 10 ng/*μ*L concentration and frozen down (-20°C) until further use. PCR assays were performed from 10 ng of cDNA in SsoAdvanced Universal SYBR Green Supermix on the CFX96™ thermocycler with the following settings: an activation step of 30 seconds at 95°C and 40 amplification cycles of denaturation (95°C for 5 sec) and annealing/extension (60°C for 30 sec). Oligonucleotide primers that were used are listed in Table 1. All samples were run in duplicate. To confirm their amplification specificity, the PCR products were subjected to a melting curve analysis. A nontemplate control was included in all experiments, and the GAPDH gene was used as endogenous control for normalization. The comparative cycle threshold (Ct) method, where the target fold = 2^−ΔΔCt^, was used to analyze the results [36]. Primary LESC cultured on TCP in 12-well plates served as the calibrator controls and had an assigned value of 1. The results were reported as a fold upregulation or fold downregulation when the fold change was greater or less than 1, respectively. Cultures from four different donor corneas were analyzed for each type of hydrogel. As cultures on hydrogels had a donor-matched culture on HAM, 6 donors were included for HAM analysis.

For statistical analysis, a linear mixed model was fitted to account for the nonindependence between observations within the same hydrogel (i.e., interdonor variation). Within this model, gene expression served as a dependent variable, the hydrogel group as an independent variable, and the donor cornea as a random intercept. The significance of the fixed effect, testing the null hypothesis that the mean outcome is the same across different culture substrates within one donor, was tested using an *F*-test with Kenward-Roger correction for the degrees of freedom. When significance of the fixed effect was observed, a post hoc analysis was carried out with a Tukey correction for multiple comparison.

## 3. Results

### 3.1. Hydrogel Production and Surface Modification

All hydrogel manufacturing protocols resulted in the successful production of hydrogels that were mechanically robust. Thickness values of collagen hydrogels which varied between groups were as follows: 133 ± 28 *μ*m for RHC I, 500 ± 50 *μ*m for CLP-12 EDC, 241 ± 98 *μ*m for CLP-12 (including CLP-12 F-*μ*CP), 303 ± 91 *μ*m for CLP-12 3D, and 244 ± 90 *μ*m for CLP-18 hydrogels. We can now confirm that thin RHC I and CLP-12 membranes can successfully undergo F-*μ*CP (Figure S1), with the quality of surface patterns being higher for CLP (75%±6) than RHC I (40%±28) hydrogels. Surface topography on CLP hydrogels was deemed successful as hydrogels remained intact and displayed a groove width close to 49 ± 2 *μ*m on bright field microscopy.

Physical characterization (Table 3; supplementary data “physical characterization of carrier membranes—Fig.  S2”) shows that water content (%) of collagen hydrogels varied between 88% and 93%, indicating that the type of collagen, type of crosslinker, and percentage of CLP did not make much of a difference. Light transmittance of collagen hydrogels was much higher than that of HAM, with values being comparable to those of native corneas. Transparency of CLP hydrogels (≥91%) was higher than that of RHC I hydrogels (84.8 ± 1.45). The refractive index of collagen hydrogels (1.34 – 1.35) was closer to that of the human cornea (1.37-1.38) compared to HAM (1.33). Permeability of the hydrogels was comparable to that of HAM, the currently used gold standard.

### 3.2. *In Vitro* Biocompatibility Testing of Hydrogels

**Figure 1:**
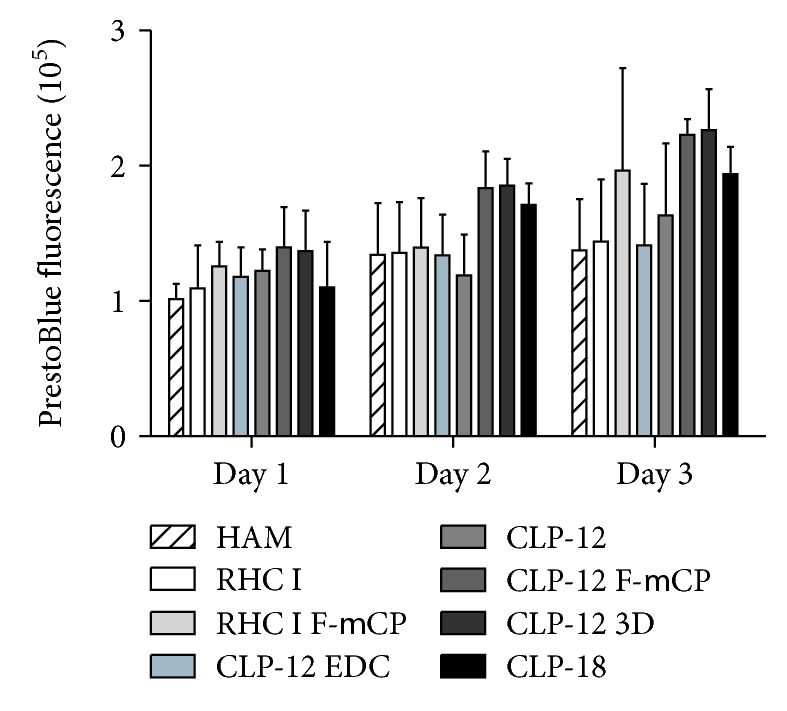
*In vitro* biocompatibility of collagen hydrogels. PrestoBlue viability assay showed that for all substrates, cumulative cell viability of iHCECs was similar at days 1, 2, and 3 of culture.

In the first set of experiments, iHCECs were cultured on different substrates and cell metabolic activity was monitored. PrestoBlue assay (Figure 1) revealed that cell metabolic activity was comparable for each of the substrates. Supplementary Live/Dead staining performed at 48 hrs postculture confirmed the biocompatibility of collagen hydrogels as cells exhibit minimal cell death on the hydrogels and comparable or lower cell death than HAM (Figure S3).

**Figure 2:**
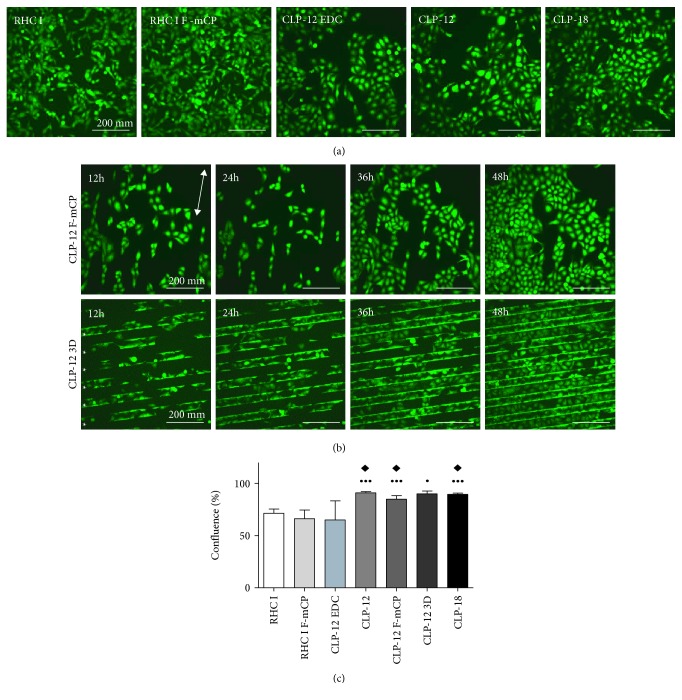
Cell growth of GFP-iHCECs on collagen hydrogels. (a) Representative images of cells cultivated for 36 hrs on RHC I, RHC I F-*μ*CP, CLP-12 EDC, CLP-12, and CLP-18 demonstrate that cells proliferate in a random pattern. (b) Micrographs of cultures on surface-modified CLP-12 F-*μ*CP and CLP-12 3D at 12 hrs, 24 hrs, 36 hrs, and 48 hrs. Cells cultured on surface-modified CLP hydrogels display a proliferation pattern that is being influenced by the fibronectin striping and 3D grooving. Orientation of F-*μ*CP stripes is shown with a double white arrow. Grooves of CLP-12 3D hydrogels are marked by white asterisk (^∗^). (c) The area of confluence at 72 hrs of culture. RHC I hydrogels displayed less confluence when compared to any of the CLP DMTMM hydrogels. RHC I F-*μ*CP showed less confluence when compared to CLP-12, CLP18%, and CLP-12 3D. ● and ●●● indicate *p* < 0.05 and *p* < 0.005, respectively, as compared to RHC I. ◆*p* < 0.05 as compared to RHC I F-*μ*CP.

Live cell imaging confirmed that both RHC I and CLP hydrogels, regardless of surface modification, supported attachment and proliferation of cells (Figure 2). Cells were found to attach to the respective hydrogel 3 hrs after seeding, which was comparable between hydrogel groups. F-*μ*CP appeared to influence cell proliferation on CLP hydrogels, unlike on RHC I hydrogels, as cells seemingly first attached to fibronectin stripes before populating the rest of the CLP hydrogel (Figure 2(b)). For CLP-12 3D, cells showed to preferentially grow first in the grooves, prior to spreading over the hydrogel's ridges (Figure 2(b)). At 72 hrs of culture, cells cultivated on CLP DMTMM hydrogels showed cell a confluence of ≥80% with an average confluence of 91.0%±1.3 for CLP-12, 85.0%±3.3 for CLP-12 F-*μ*CP, 90.0%±2.7 for CLP-12 3D, and 89.6%±1.2 for CLP-18 (Figure 2(c)). The average confluence of RHC I and RHC I F-*μ*CP was 71.4%±4.1 and 66.27%±8.3, respectively, with RHC I showing significantly less confluence compared to any of CLP DMTMM hydrogels and RHC I F-*μ*CP being less confluent then CLP-12, CLP-12 3D, and CLP-18 (Figure 2(c)). The lowest average confluence was observed for CLP-12 EDC (65.1%±18.3), with 2 sites showing a confluence of <10% (data not shown).

**Figure 3:**
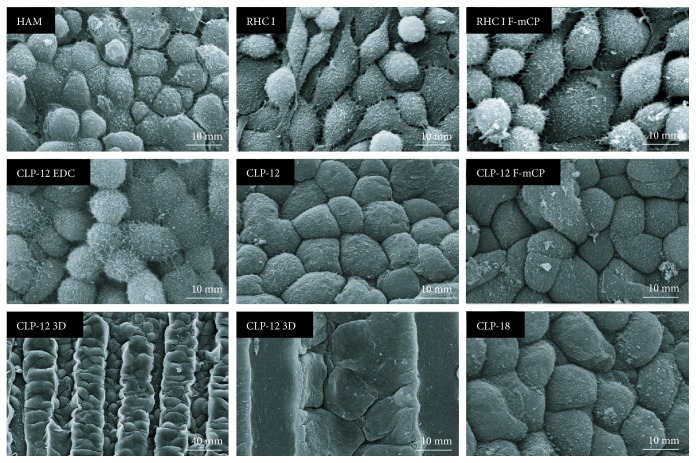
SEM imaging of iHCECs cultivated on HAM and collagen hydrogels for 4 days. Cells had formed a confluent monolayer. In general, cells exhibited a typical cobblestone appearance. At a region where cells had not reached full confluence on CLP-12 3D (lower middle), cells were mainly present in the grooves.

**Figure 4:**
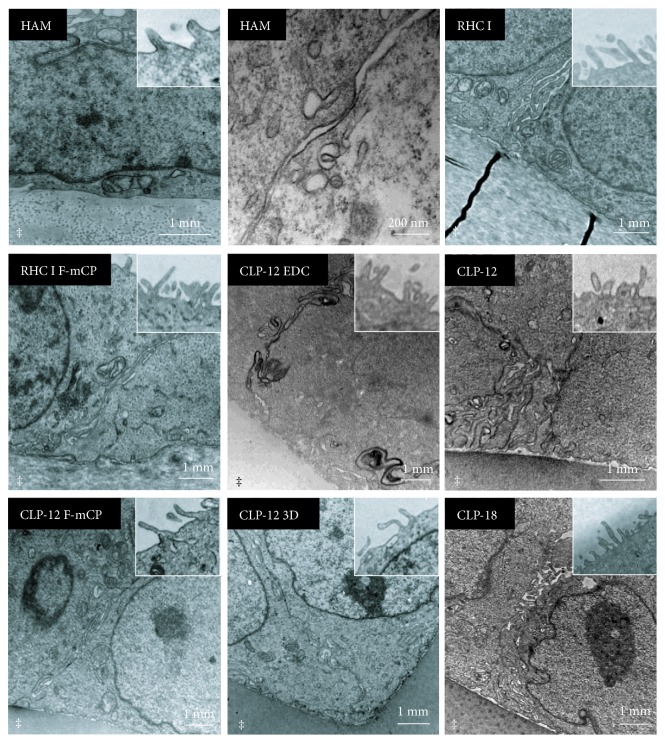
TEM micrographs of iHCECs on different substrates after 4 days of cultivation. A confluent monolayer had formed, in which cells displayed multiple interdigitations and apical microvilli (inset). Cells cultivated on HAM displayed expression of gap junctions (middle top), whereas cells cultivated on collagen hydrogels had not initiated differentiation as desmosomes, hemidesmosomes, and gap junctions could not be observed. ‡ denotes the carrier material.

After 4 days of cultivation, SEM imaging (Figure 3) was performed at regions that had reached full confluence. Cultured cells displayed the typical cobblestone appearance; however, at RHC I hydrogels, some isolated cells displayed an elongated morphology. In a region where cells had not reached full confluence on CLP-12 3D hydrogels, cells were mainly observed in the grooves and not on the ridges of the hydrogel. TEM imaging (Figure 4) of cultures confirmed that a monolayer of cells had formed on all substrates and that cells displayed apical microvilli. Furthermore, it was noted that cells cultivated on collagen hydrogels had not initiated differentiation, whereas cells cultivated on HAM expressed gap junctions in the absence of stratification, indicating early differentiation.

### 3.3. Cultivation and Characterization of Primary LESC Cultures

**Figure 5:**
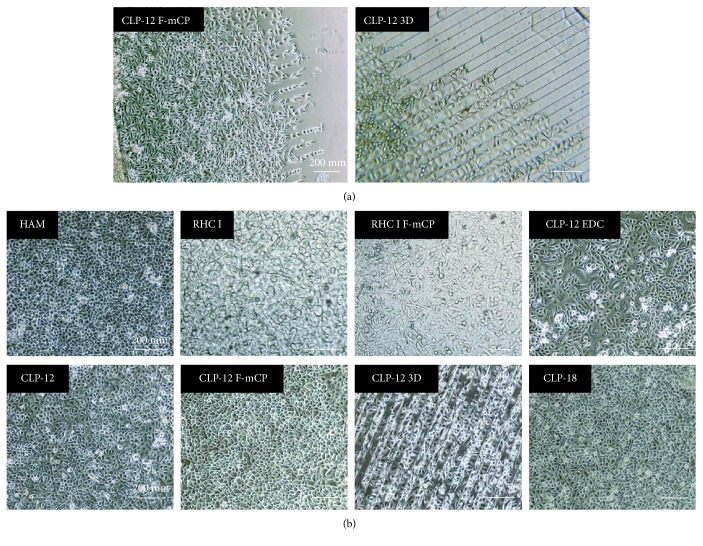
Representative images of primary limbal epithelial cell cultures at day 4 (a) and day 14 (b) on various carrier materials. At day 4, it is evident that surface modification of CLP hydrogels influences limbal epithelial cell outgrowth, as cell first grew on fibronectin stripes or in grooves, before spreading over the rest of the hydrogel growth. By day 14, cell confluence was reached in a 15 mm diameter outgrowth on both types of RHC I gel, all types of CLP DMTMM gel, and HAM. After 14 days, cells on CLP-12 EDC had not reached full confluence.

A total of 41 eyes of 22 donors, with an average donor age of 73.7 ± 11.3 years (range 49-90 years), were used for this study. Cultured epithelial cells were analyzed every 2 days with phase contrast microscopy (Figure 5). By day 3 of culture, epithelial cells had emerged from 83% of limbal biopsies. Explants that did not prove successful by day 3 did not display cell outgrowth later. No significant difference for successful initiation of explant cultivation was observed between different substrates (data not shown). During the first week of cultivation, cells that were cultivated on the surface of the modified CLP hydrogels displayed a distinctive proliferation pattern (Figure 5(a)). In accordance with the observations made at live cell imaging, pioneer cells followed fibronectin patterns, colonizing the intermediate area in the following hours and days. Similarly, cells on 3D hydrogels grew first in the grooves before expanding over the ridges. On day 14 of culture (Figure 5(b)), outgrowth on all substrates contained small and cuboidal epithelial-like cells of varying cell size. Cells cultured on HAM maintained a smaller round shape compared to cells cultivated on hydrogels. In contrast, cells cultured on RHC I and RHC I F-*μ*CP displayed a more heterogeneous morphology with singular elongated cells being observed in between simple squamous epithelial cell growth. By day 14, all cultures on CLP DMTMM hydrogels had reached near confluence on the 15 mm diameter gel. In contrast, none of the CLP-12 EDC hydrogels reached confluence; moreover, 3 out of 9 cultures generated too low cell yield for further characterization and 5 out of 9 cultures did not meet the minimum 8 mm diameter outgrowth, which is deemed a quality standard at our center. At day 14, RHC I and RHC I F-*μ*CP cultures displayed an average cellular outgrowth of >15 mm, whereas HAM cultures had reached subconfluence in a 14-15 mm diameter.

#### 3.3.1. Immunohistochemical Characterization

**Figure 6:**
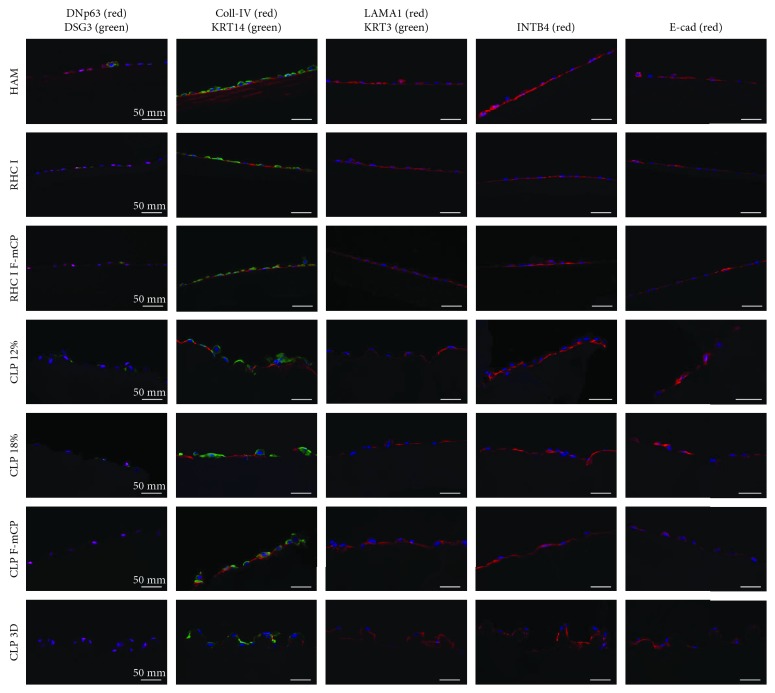
Immunohistochemistry of primary limbal epithelial cells cultured on different carrier materials. Representative images of double immunostaining for detection of ΔNp63-DSG3, laminin-KRT3, and Coll-IV-KRT14 and monostaining for detection of INTB4 and E-cad. Overall, cultivated cells show high expression of (i) ΔNp63, a nuclear stem cell marker, (ii) KRT14, a cytoplasmic proliferation marker, and (iii) laminin, Coll-IV, INTB4, and E-cad, markers of corneolimbal basal cell adhesion molecules and LESC niche-related extracellular matrix. Low expression of proliferation markers DSG3 and KRT3 indicates that cultivated primary cells exhibit a LESC phenotype. All stains were negative in hydrogel-only (no cell) control samples (data not shown).

*(1) Expression of Stem Cell Markers*. The stemness of cultivated cells was verified with ΔNp63 and KRT14 (Figure 6). Both markers have been attributed to progenitor epithelial cells in the basal and suprabasal layers of the limbus [38–40]. Cells cultivated on any of the substrates showed nuclear staining of ΔNp63 and cytoplasmic staining of KRT14. No immediate difference in the pattern of expression was observed between different substrates.

*(2) Expression of Differentiation Markers*. KRT3 and DSG3 were used as differentiation markers for corneal epithelial cells. KRT3 is a cytoplasmic keratin that has been shown to be specific for corneal epithelium [41], and DSG3 is a glycoprotein component of desmosomes [42, 43]; both markers display differentiation-related expression. Cultivated cells showed low expression of both markers, with only few isolated cells that were KRT3 or DSG3 positive. Double staining with ΔNp63 confirmed that DSG3-positive cells lacked expression of the stem cell marker (Figure 6—HAM). Pattern of expression was comparable between tested scaffolds.

*(3) Expression of Extracellular Matrix Proteins and Cell Adhesion Markers*. Laminin and Coll-IV have been described as extracellular matrix and basement membrane components of the human cornea and limbus [42, 44]. Extracellular expression of both markers was noted, indicating deposition of laminin and Coll-IV by cultivated cells on the respective substrate (Figure 6). Coll-IV also is a key component of HAM [45], which is shown by the Coll-IV-positive staining of HAM stroma (Figure 6). INTB4 and E-cad have been proposed to mediate cell anchorage of basal epithelial cells, with INTB4 expression being confined to LESC [43, 46] and E-cadherin expression being more specific for basal and suprabasal corneal epithelial cells [47]. Both markers show positive membrane expression in cultivated cells. INTB4 expression appears to be confined to the basal side, whereas E-cad expression is more diffuse and is expressed in the cytoplasm and at the basal and apical sides. No apparent difference in the pattern of expression was noted between the tested substrates.

#### 3.3.2. Gene Expression of Limbal Epithelial and Corneal Epithelial Cell Makers

**Figure 7:**
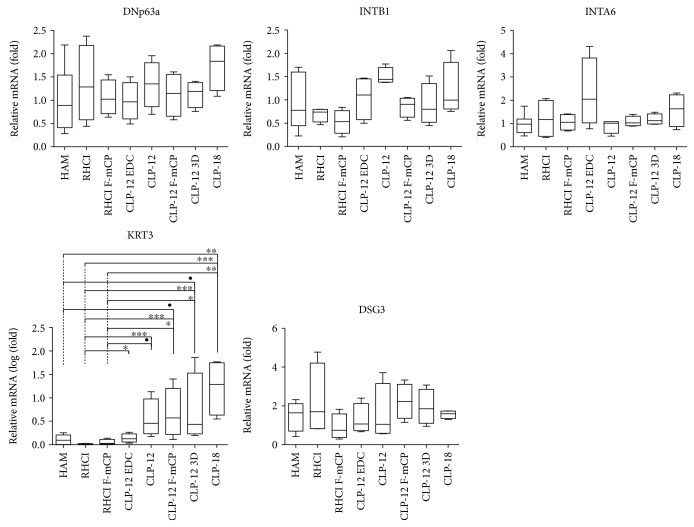
Relative gene expression of stem cell markers (ΔNp63*α*), adhesion markers (INTB1, INTA6), and differentiation markers (KRT3, DSG3) of primary limbal epithelial cells cultured on different carrier materials. Statistical significance (*p* ≤ 0.05) and statistical trends (*p* ≤ 0.1) were noted only for KRT3 expression. •*p* ≤ 0.1; ^∗^ *p* ≤ 0.05; ^∗∗^ *p* ≤ 0.01; ^∗∗∗^ *p* ≤ 0.001.

With the house keeping gene, GAPDH, as an internal control and cell cultures on TCP as a calibrator, RT-qPCR showed positive expression for ΔNp63*α*, KRT3, DSG3, INTB1, and INTA6 (Figure 7). Integrin-*β*1 and integrin-*α*6 are known to be progenitor cell-specific adhesion proteins [43]. A statistically significant difference between groups was only observed for KRT3 expression (Figure 7), with RHC I, RHC I F-*μ*CP, and HAM showing significantly lower expression levels compared to CLP hydrogels. More specifically, significantly lower KRT3 expression was noted for RHC I when compared to any of the CLP hydrogels, whereas lower expression was noted for RHC I F-*μ*CP when compared to CLP-12 F-*μ*CP, CLP-12 3D, and CLP-18 and for HAM when compared to CLP-18. Furthermore, a trend (*p* ≤ 0.1) was observed for lower KRT3 expression in HAM than in CLP-12 F-*μ*CP and CLP-12 3D and in RHC I F-*μ*CP than in CLP-12. The relative fold change in gene expression is listed as supplementary data (Table S2).

## 4. Discussion

CLET has been a major breakthrough for the treatment of patients suffering from LSCD [4, 5, 14]. However, protocols for LESC cultivation need further optimization and standardization as they commonly involve animal-derived supplements, such as growth factors, serum, and/or fibroblast feeder layers [48], all of which could induce zoonosis, allergy, and other side effects [49]. In the context of GMP regulations, both stem cell carrier and culture protocol should meet strict guidelines in quality. Attempts have been made to remove xenogeneic products from culture protocols [5, 50, 51] in order to achieve standardization. Standardizing HAM, the carrier material most frequently used in CLET, remains challenging as it is characterized by considerable variation in physicochemical properties [10–13].

CLP and RHC I both originate from xeno-free sources and are optically transparent, mechanically stable, and biocompatible *in vivo* [27, 28]. CLP-12 EDC hydrogels have shown great promise as an acellular corneal construct *in vivo* [27, 28]. However, EDC/NHS crosslinking is too fast to allow successful casting and production of thin hydrogels (<300 *μ*m thick) [52]. To generate a flexible and thin scaffold, we used DMTMM as an alternative crosslinker. Other groups have demonstrated the efficiency of DMTMM over EDC/NHS for crosslinking of peptides and glycosaminoglycans as DMTMM resulted in slower crosslinking time and hence more homogenous hydrogels [53]. Similar to EDC/NHS, DMTMM is a zero-length crosslinker, implying that it is not incorporated into the scaffold. DMTMM has the advantage of not requiring pH control or cause pH shift during the reaction over EDC/NHS, ensuring good reaction yields and biocompatibility [53, 54]. In this study, we used DMTMM to crosslink collagen derivatives [55] and tested its *in vitro* performance. RHC I was not crosslinked with DMTMM since our previous research demonstrated that EDC/NHS-crosslinked RHC I gels were of desired thickness (<150 *μ*m thick) and flexibility.

Our data demonstrate that RHC I and CLP hydrogels, irrespective of type of crosslinker, support cultivation of iHCECs and primary limbal epithelial cells. CLP-12 EDC, however, was the only tested carrier material for which primary limbal epithelial cultures did not meet quality standards due to low cell yield. Interestingly, our previous data indicate that CLP-12 EDC might be a suitable acellular alternative to conventional corneal transplantion [27]. In the work of Jangamreddy et al., we noted that suboptimal growth of iHCECs at days 1 and 2 on CLP-12 EDC hydrogels does not necessarily translate into inferior *in vivo* performance [27]. After all, acellular CLP-12 EDC corneas supported functional reepithelialization when implanted in pigs. Moreover, CLP implants performed equally with RHC III-based hydrogels, of which the latter have successfully been implanted in humans [18, 20]. Nonetheless, we have now shown that CLP-12 EDC is less suitable for standardized *in vitro* cultivation of primary limbal epithelium. Conversely, RHC I hydrogels did meet quality standards of cell outgrowth for primary limbal cultures, although significant lower confluence was observed for iHCEC cultures compared to CLP DMTMM hydrogels after 3 days of cultivation. This discrepancy might be explained by the fact that iHCECs display a significantly altered genomic profile to primary limbal epithelium [56]. This must be carefully considered when drawing conclusions on data obtained through their use.

In our experiments, surface modification of RHC I did not influence pattern of cell proliferation, which might be attributed to the inherent biocompatibility of RHC [18–21, 27, 31, 32, 57, 58]. Even though surface modification of CLP DMTMM hydrogels influenced the pattern of cell proliferation, the rate of outgrowth and the genotype and phenotype of cultured cells were not significantly altered. It can therefore be assumed that surface patterning of RHC I and CLP DMTMM hydrogels may be unnecessary to attain successful LESC-enriched cultures *in vitro*. Our results confirm the findings of Hogerheyde et al. [59], in which fibronectin coating of silk fibroin membranes did not significantly improve cell outgrowth of iHCECs or of primary LESC. Conversely, our results are in contradiction to previous research that showed the benefit of fibronectin patterning of carrier membranes on cell proliferation of iHCECs [21, 60]. In the study of Islam et al., a similar fibronectin patterning protocol was used as that in our study; however, the membrane of interest was RHC III-MPC and anti-Ki67, anti-focal adhesion kinase, and anti-integrin-*β*1 were used as antibodies in immunohistochemical characterization [21].

In 2013, the group of Levis et al. introduced RAFT (Real Architecture for 3D Tissue) hydrogels, a collagen construct produced through plastic compression of bovine type I collagen, as an *in vitro* model of the human cornea [61]. To mimic limbal crypts, 3D grooves were created in the hydrogel's surface (RAFT TE). RAFT TE successfully support limbal epithelium cultivation; however, clinical impact is limited since RAFT lacks chemical crosslinking and thus results in heterogeneous hydrogels with suboptimal optical and mechanical properties [61–63]. Furthermore, biocompatibility of RAFT gels has yet to be validated *in vivo.*

When compared to our study, aforementioned reports [21, 59–62] lacked (i) extensive phenotyping and genotyping of cultured primary cells, (ii) comparison to HAM, the carrier of choice in CLET, and/or (iii) implementation of a xeno-free, standardized cultivation protocol; all of which indicate further need of optimization-described techniques.

To the best of our knowledge, we are the first to cultivate primary human limbal epithelium on collagen hydrogels using a xeno-free and fully standardized culture protocol. The CnT-PR medium is a GMP-grade culture medium that has been selectively developed to target progenitor cells of epithelial lineage and to inhibit proliferation of cells from mesenchymal lineage [50, 64]. By using a GMP-grade culture medium, the proposed cultivation protocol could be translated into a clinical setting with relative ease. In our setup, primary cells grew to confluence within 14 days of culture, except for CLP-12 EDC, without the addition of 3T3 feeder layers or xenobiotics. RHC I hydrogels could only successfully support primary cells when 1% hAB was added to the culture medium. For CLP hydrogels, a serum-free culture medium was attained but hydrogels had to be soaked for at least 4 days in culture medium to consistently demonstrate explant outgrowth (data not shown).

Immunostaining revealed that cultured cells show low expression of differentiation markers KRT3 and DSG3 and high expression of progenitor marker ΔNp63, proliferation marker KRT14, and adhesion markers INTB4 and E-cad. Furthermore, cells deposited extracellular matrix and basement membrane proteins laminin and Coll-IV. This pattern of protein expression strongly suggests that cells cultured on any of the carriers can be identified as LESC [38–43, 46, 65]. E-cad expression remained largely cytoplasmic, with cell membrane localization to a certain extent. This pattern of staining shows that E-cad expression had not reached a maximum, indicating that cells were in a relatively undifferentiated state. *In vitro* supplementation with CaCl_2_ (Figure S4) shows that E-cad expression is located mainly at the cell membrane when cells are initiating differentiation. Even though a nonspecific ΔNp63 antibody was used for immunohistochemistry that targeted all three ΔNp63 isoforms (*α*, *β*, and *γ*), previous work from Di Iorio et al. [39] indicated that LESC strictly contain the *α*-isoform and not *β-* or *γ*-isoforms. Differentiated corneal epithelium on the other hand does not contain any of the isoforms. In PCR analysis, a specific ΔNp63*α* primer was used.

Apart from KRT3, other markers that were targeted with RT-PCR have shown comparable expression between different groups. In general, a significant difference in relative expression of KRT3 between CLP and RHC I hydrogels was observed, with the RHC group showing lower expression. KRT3 expression levels for CLP hydrogels are well below levels observed for corneal epithelium (>2500; data not shown) or *in vitro*-differentiated limbal epithelial cells (Figure S4). Placing gene expression patterns into the perspective of differentiated cells indicates that all hydrogels perform extremely well, with RHC I slightly outperforming CLP DMTMM. It is not clear whether lower KRT3 expression is inherent to RHC I hydrogels or must be attributed to the adjusted culture protocol that involves 1% hAB supplementation. Both our results and the data from González et al. indicate that the concentration of hAB, more than the carrier material, might influence the level of differentiation [51]. Finally, it should be noted that KRT3 mRNA expression is relatively low (Cq value: 27-38) compared to the other investigated markers (Cq: 21-27). Therefore, mRNA expression might not be present in sufficient quantities to promote protein expression, hence, KRT3-negative IHC staining in LESC cultures. Data obtained through *in vitro* differentiation confirm this theory, as mRNA expression of KRT3 resulted in KRT3 protein detection (Figure S4).

Both primary cells and iHCECs were cultured in the absence of airlifting and CaCl_2_ supplementation and therefore did not initiate differentiation nor stratification [66–68]. This may be regarded as a limitation of our study since most methods used to establish limbal epithelial cultures favor terminal differentiation over preservation of stemness [69–71]. However, for long-term restoration of the damaged ocular surface, preservation of LESC population may be required during the culture process and postgrafting [4, 39, 51, 66–68, 72–74]. Previous research using an *in vitro* RAFT TE model concluded that airlifting was not required to maintain a functional epithelium on collagen hydrogels and that a higher yield of ΔNp63*α*-positive cells was obtained in nonairlifted cultures [74]. In our study, we provide evidence that cells cultivated in CnT-PR successfully sustained their undifferentiated LESC state, not only on HAM [51] but also on collagen-based hydrogels. Furthermore, cells cultivated in CnT-PR maintain their ability to initiate differentiation, simply by adding 1.1 mM CaCl_2_ to the culture medium (Figure S4).

Finally, it should be stressed that collagen-based hydrogels are highly tunable, creating opportunities previously unseen in CLET. Firstly, the thickness of collagen hydrogels can be adjusted to tackle deeper corneal disease and thus reduce the need for secondary corneal transplantation post-CLET. This would be a considerable advantage over other carrier materials such as HAM, silk fibroin, siloxane hydrogels, and fibrin-coated contact lenses [4, 5, 75, 76]. Secondly, supporting niche cells such as limbal MSCs and melanocytes could be incorporated into collagen hydrogels to allow coculture of niche-related cells. Cocultures could play an important role in the maintenance of a vast LESC side population through improved mimicry of the native stem cell niche [74, 77]. However, long-term survival of supporting niche cells and possible clinical benefit remain to be validated *in vivo*. Thirdly, collagen hydrogels offer additional opportunities of surface patterning, which is a fully customizable process. Even though our results did not indicate a short-term benefit for *in vitro* cultivation of LESC, surface patterning might potentially result in an ideal microenvironment for long-term LESC proliferation and preservation. In addition to F-*μ*CP and 3D fabrication, other groups have suggested surface tethering and bulk incorporation of laminin, collagen type III, Coll-IV, IKVAV, YIGSR, RGD, and vitronectin [46, 59, 78, 79]. All of these possibilities support the promise of collagen hydrogels in tissue engineering, not only in ophthalmology but also in other disciplines such orthopedics [80], dermatology [81], and cardiology [82].

## 5. Conclusion

Based on our findings, we conclude that RHC I and CLP hydrogels successfully support *in vitro* cultivation of iHCECs and primary LESC. When compared to HAM, primary cell cultivation on RHC I and CLP DMTMM hydrogels showed comparable (i) cell outgrowth and (ii) ΔNp63*α*-positive cell yield. We provide evidence that surface patterning, through 3D molding or F-*μ*CP, influences cell attachment and cell proliferation for CLP DMTMM hydrogels but not for RHC I hydrogels. Our results indicate that surface patterning does not impact the cell phenotype or genotype, but it could be that the clinical significance of surface patterning may only become apparent in an *in vivo* setting. Finally, for reasons unknown to us, CLP-12 EDC hydrogels resulted in suboptimal primary cell cultivation and underperformed when compared to the other tested carriers. In conclusion, RHC I and CLP DMTMM show promise in the cultivation of LESC and contribute to the development of a culture protocol in which both the carrier material and culturing technique are xeno-free and fully standardized.

## Figures and Tables

**Table 1:** Oligonucleotide primers and primers used for reverse transcriptase PCR.

Gene name	Gene symbol	Assay ID
Glyceraldehyde-3-phosphate dehydrogenase	GAPDH	qHsaCED0042632
*β*-2-Microglobulin	B2M	qHsaCED0015347
ΔNp63*α* [37]	ΔNp63*α*	Fw: GCATTGTCAGTTTCTTAGCGAG
		Rev: CCATGGAGTAATGCTCAATCTG
Cytokeratin 3	KRT3	qHsaCID0005917
Desmoglein 3	DSG3	qHsaCID0015226
Integrin-*β*1	INTB1	qHsaCED0005248
Integrin-*α*6	INTA6	qHsaCED0042632

**Table 2:** Tested carrier material.

Abbreviation	Material	Crosslinker	Surface patterning	Collagen concentration^∗^
HAM	Denuded HAM	—	—	—
RHC I	RHC I	EDC/NHS	—	~3 mg/cm^2^
RHC I F-*μ*CP	RHC I	EDC/NHS	Fibronectin microcontact printing	~3 mg/cm^2^
CLP-12 EDC	CLP-PEG	EDC/NHS	—	12%
CLP-12	CLP-PEG	DMTMM	—	12%
CLP-12F-*μ*CP	CLP-PEG	DMTMM	Fibronectin microcontact printing	12%
CLP-12 3D	CLP-PEG	DMTMM	3D topography	12%
CLP-18	CLP-PEG	DMTMM	—	18%

^∗^Concentration of RHC I is expressed as net weight at collagen casting (mg/cm^2^). Concentration of CLP is expressed as percentage of stock solute (%). —: not applicable; HAM: human amniotic membrane; RHC I: recombinant human collagen type I; CLP-PEG: PEGylated collagen-like peptide; EDC: 1-ethyl-3-(3-dimethyl aminopropyl) carbodiimide; NHS: N-hydroxysuccinimide; DMTMM: 4-(4,6-dimethoxy-1,3,5-triazin-2-yl)-4-methyl-morpholinium chloride.

**Table 3:** Properties of RHC I, CLP-12 EDC, and CLP DMTMM hydrogels, with HAM and the human cornea serving as control.

Properties	HAM	RHC I	CLP-12 EDC	CLP-12	CLP-18	Human cornea
Water content (%)	87.68 ± 0.02	89.21 ± 0.01	91.65 ± 1.10 [28]	90.31 ± 0.02	88.42 ± 0.01	78 [39]
Refractive index	1.33	1.35	1.34 [27]	1.35	1.35	1.37-1.38 [40]
Transmission at 490 nm (%)	60.0 ± 2.2	84.8 ± 1.5	92.4 ± 1.0 [28]	91.0 ± 0.8	91.0 ± 0.5	87.1 ± 2.0 [41]
Apparent permeability (*P*_app_) (cm/sec)	0.039 ± 0.007	0.057 ± 0.03	0.061 ± 0.003	0.056 ± 0.006	0.047 ± 0.006	NA

## Data Availability

The data used to support the findings of this study are available from the corresponding author upon request.

## Supplementary Materials

Supplementary 1 Figure S1: surface modification of RHC I and DMTMM-crosslinked CLP-12 hydrogels. Fluorescence micrograph of RHC I (a) and CLP (b) hydrogel that have been surface modified with 488 nm fluorescent fibronectin. SEM image (c) of CLP-12 3D hydrogels with grooves that are 50 *μ*m wide and 20 *μ*m deep.

Supplementary 2 “Physical characterization of carrier membranes—Fig. S2”: methodology and results of physical characterization of tested carrier materials. Figure S2 is an illustration of the cumulative permeability of the tested collagen hydrogels and HAM.

Supplementary 3 Figure S3: Live/Dead staining of iHCECs cultured on various carrier membranes.

Supplementary 4 Figure S4: characterization of in vitro differentiation of primary limbal epithelial cells.

Supplementary 5 Table S1: antibodies used in immunohistochemical analyses.

Supplementary 6 Table S2: relative fold change in gene expression of limbal epithelium cultivated on different carrier materials.
