# Supplementary material for: In Vitro Cultivation of Limbal Epithelial Stem Cells on Surface-Modified Crosslinked Collagen Scaffolds
Source: Stem Cells Int. 2019 Apr 1;2019:7867613. doi: 10.1155/2019/7867613 (PMC6466865; doi:10.1155/2019/7867613)
Supplement: Supplementary 1 — Figure S1: surface modification of RHC I and DMTMM-crosslinked CLP-12 hydrogels. Fluorescence micrograph of RHC I (a) and CLP (b) hydrogel that have been surface modified with 488 nm fluorescent fibronectin. SEM image (c) of CLP-12 3D hydrogels with grooves that are 50 μm wide and 20 μm deep. [file 7867613.f1.docx]

### Fig. S1: Surface modification of RHC I and CLP hydrogels


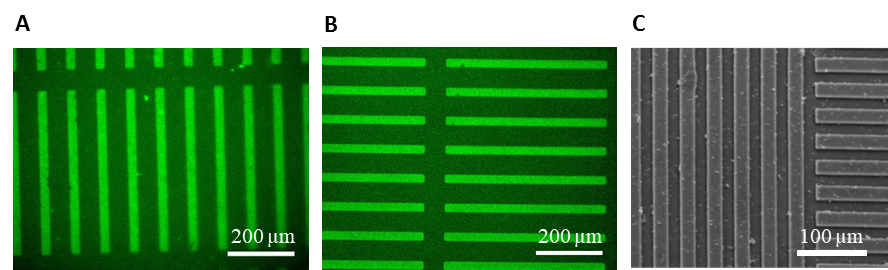


**Fig. S1.** Surface modification of RHC I and DMTMM-crosslinked CLP-12 hydrogels. Fluorescence micrograph of RHC I **(A)** and CLP **(B)** hydrogel that have been surface modified with 488 nm fluorescent fibronectin. Fibronectin stripes were successfully printed on both RHC I and CLP hydrogels, with striping diameter averaging 30 µm width and 60 µm spacing. SEM image **(C)** of CLP-12 3D hydrogels with grooves that are 50 µm wide and 20 µm deep, interspersed with ridges of the same dimension. At sample preparation for SEM, CLP-12 hydrogels showed extensive shrinkage as width of the grooves had shrunk to 17 ± 1 µm, and ridges had shrunk to 22 ±1 µm due to sample dehydration and critical point drying.
