# Supplementary material for: In Vitro Cultivation of Limbal Epithelial Stem Cells on Surface-Modified Crosslinked Collagen Scaffolds
Source: Stem Cells Int. 2019 Apr 1;2019:7867613. doi: 10.1155/2019/7867613 (PMC6466865; doi:10.1155/2019/7867613)
Supplement: Supplementary 2 — “Physical characterization of carrier membranes—Fig. S2”: methodology and results of physical characterization of tested carrier materials. Figure S2 is an illustration of the cumulative permeability of the tested collagen hydrogels and HAM. [file 7867613.f2.docx]

### Physical characterization of carrier membranes – Fig. S2

***Methodology***

To measure water content, samples were DW-equilibrated for 24 hrs. Water content of samples was determined by weighing membranes that were blotted dry to remove excess surface liquid to obtain the wet weight (W_0_). These samples were then dried in a drying oven (60°C) for 24 hrs to obtain the dry weight (W). Equilibrated water content of hydrogels (W_t_%) was obtained according to the following equation: W_t_% = (W_0_ – W)/W_0_ x 100%.

Refractive index (RI) of samples was measured of fully hydrated membranes using an Abbe refractometer (Model C10, VEE GEE Scientific Inc., Kirkland, WA, USA) at 19°C with DW as the calibration agent. To test membrane light transmission, 6 mm acellular discs of the sample were punched out and placed in a glass bottom 96-well plate. Using the previously published protocol [35], light absorbance was measured at room temperature for specific wavelengths of the visual spectrum (490 nm) with the VICTOR^3^ microplate reader (PerkinElmer). Percentage of light transmittance was calculated with following equation: Transmission (%) = 10^(2 - Absorbance)^. Wells mounted with 100 µL of Trypan Blue or DW served as negative and positive controls, respectively.

The permeability of HAM and the hydrogel cell carriers was investigated by measuring the diffusion of small (700 Da) fluorescent marker Alexa Fluor® 568 hydrazide sodium salt (Life Technologies, Thermo Fisher Scientific), at a concentration of 10 µM. The permeability study was conducted with the Ussing Chamber system (Physiologic instruments, San Diego, CA) with P2300 EasyMount Diffusion Chambers and P2307 sliders. Approximately 6 mm x 6 mm samples were cut from HAM and RHC I and clamped between the sliders, whereas for the more fragile CLP-hydrogels, samples with a diameter of 10 mm and thickness of 500 µm were used. The diffusion of the fluorescent marker through the materials was studied by adding equal volumes (2.1 ml) of PBS with fluorescent marker to the donor chamber and pure PBS to the receiving chamber. Two 100 µl samples were drawn from the receiving chamber at time points 60, 120, 180, 240, 300, 360 and 420 min, and replaced with fresh PBS. Fluorescence of the samples was measured using Wallac Viktor2 1420 Multilabel counter (PerkinElmer) at 590 nm excitation and 642 nm emission wavelengths. The permeability of each sample was characterized by calculating the apparent permeability coefficient (P_app_, cm s^-1^) with the equation P_app_ = (dC/dt)/(60C_0_A), where dC/dt is the slope, C_0_ the initial concentration of the donor chamber and A the exposed surface area of the sample in the slider (0.031 cm^2^). Additionally, cumulative permeability of the materials was calculated as the percentage of diffused fluorescent marker from the donor chamber to the receiving chamber during the experiment. Permeability

measurements were conducted for three to four parallel samples for each carrier material.

***Results: Permeability testing***

Permeability of the hydrogels (Fig. S2) to the 700 Da fluorescent marker were comparable to that of HAM, the currently used gold standard. Although the apparent permeability coefficients between the different carrier materials did not show significant differences, RHC I had almost 10-fold standard deviation in P_app_ compared to the other materials, possibly indicating larger variance in the hydrogel structure. The cumulative permeability (Fig. S2), indicates that there is a slight difference in the permeability behavior of the biological HAM compared to the other carrier materials. The cumulative permeability for the hydrogels remained approximately linear for the duration of the experiment, whereas the cumulative permeability of HAM plateaued after six hours.

Fig. S2. The cumulative permeability of the different carrier materials for a 700 Da fluorescent marker. Cumulative permeability for the hydrogels remained approximately linear for the duration of the experiment, whereas the cumulative permeability of HAM plateaued after six hours.
