# Supplementary material for: In Vitro Cultivation of Limbal Epithelial Stem Cells on Surface-Modified Crosslinked Collagen Scaffolds
Source: Stem Cells Int. 2019 Apr 1;2019:7867613. doi: 10.1155/2019/7867613 (PMC6466865; doi:10.1155/2019/7867613)
Supplement: Supplementary 3 — Figure S3: Live/Dead staining of iHCECs cultured on various carrier membranes. [file 7867613.f3.docx]

### Fig. S3: Live/Dead staining of iHCECs cultured on various carrier membranes


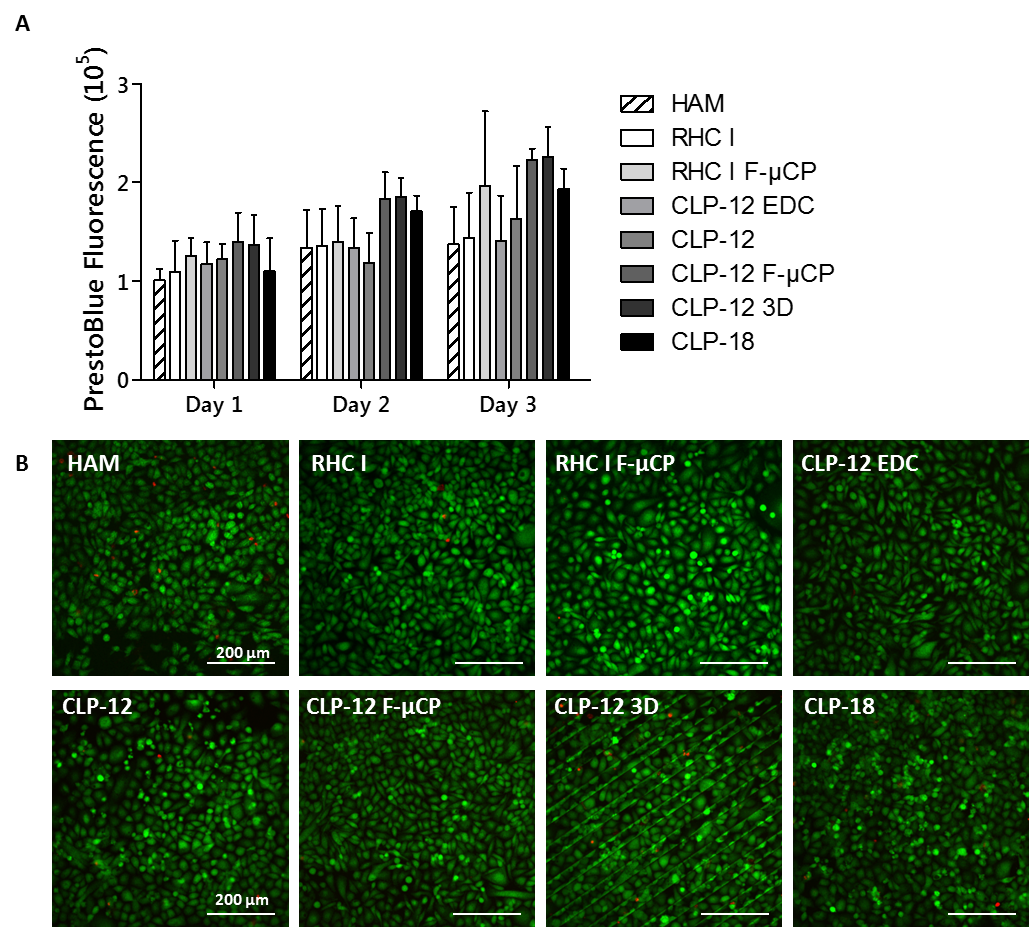


**Fig. S3.** *In vitro* biocompatibility of collagen hydrogels. Live/Dead staining shows live cells as green, while dead cells fluoresce red. This assay confirmed the biocompatibility of collagen hydrogels as cells exhibit minimal cell death on the hydrogels.
