# Supplementary material for: In Vitro Cultivation of Limbal Epithelial Stem Cells on Surface-Modified Crosslinked Collagen Scaffolds
Source: Stem Cells Int. 2019 Apr 1;2019:7867613. doi: 10.1155/2019/7867613 (PMC6466865; doi:10.1155/2019/7867613)
Supplement: Supplementary 4 — Figure S4: characterization of in vitro differentiation of primary limbal epithelial cells. [file 7867613.f4.docx]

### Fig. S4: In vitro differentiation of LESC


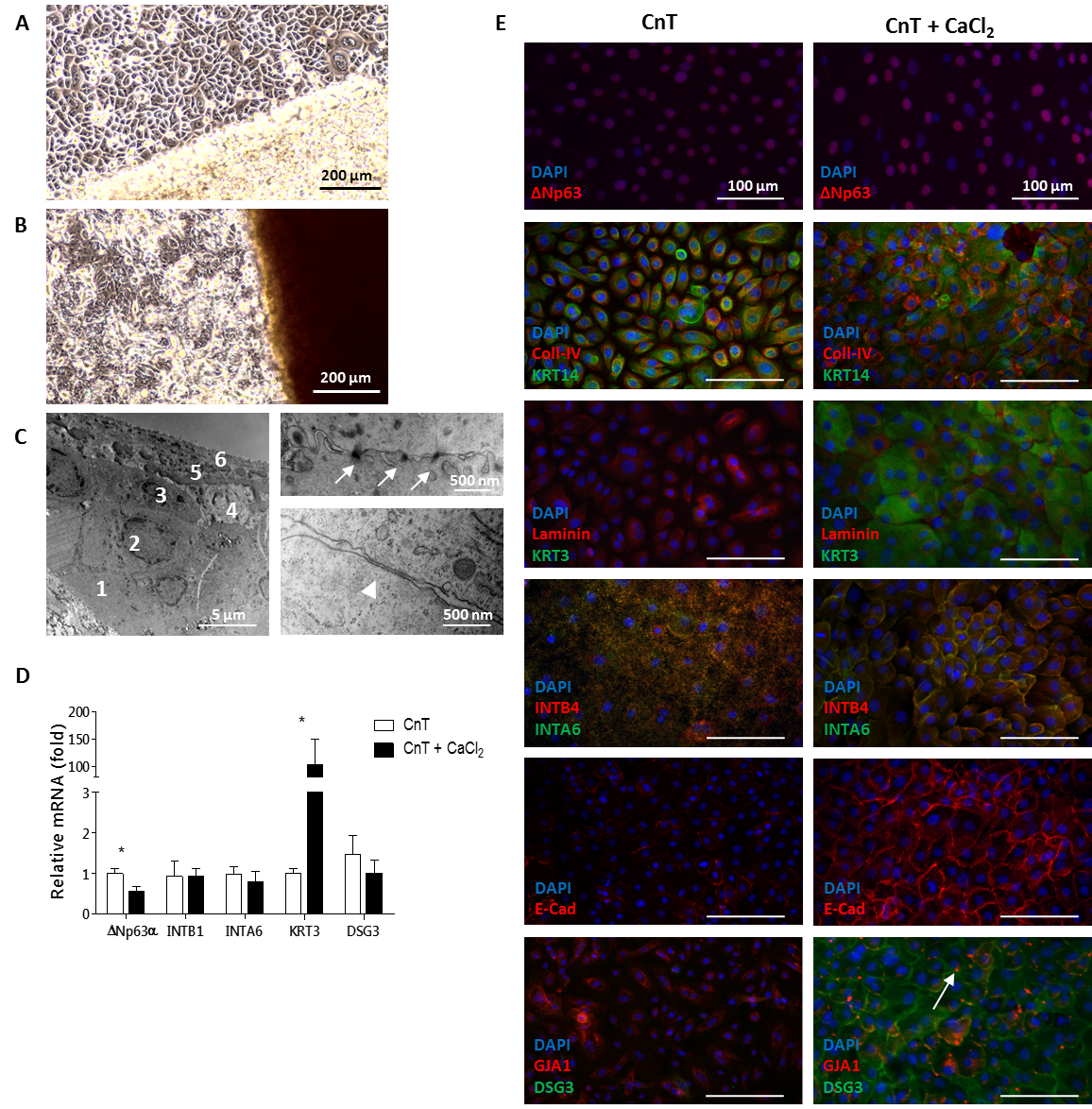


**Fig. S4.** *In vitro* differentiation of primary limbal epithelial cells. Limbal explants were cultivated on TCP for 10 days in CnT-PR, after which 1.1 mM CaCl_2_ (VWR) was added to the culture medium, and cells were cultured 8 more days. Brightfield micrographs at day 14 reveal cell morphology of CnT-PR cultures **(A)** and CnT-PR + CaCl_2_ cultures **(B)**. Cells cultured in CnT-PR displayed the typical cobblestone morphology of small cells that formed a monolayer, whereas cells supplemented with CaCl_2_ displayed multilayering of elongated squamous cells. **C.** TEM imaging reveals that stratification occurred in 5-6 layers and that desmosomes (arrow) and gap junctions (arrowhead) had formed as intercellular connections. **D.** RT-PCR analysis confirms that cells cultured in CnT-PR + CaCl_2_ had initiated differentiation, with differentiated cells expressing significant higher levels of KRT3 and lower levels of ΔNp63α (* p≤0.05). Interestingly, DSG3, another differentiation-related marker, was not upregulated, possibly indicating that cells had reached maximal expression of desmosomes, and DSG3 mRNA levels had fallen to baseline values to maintain steady desmosome renewal. **E.** Immunostaining reveals that cells cultured in CaCl_2_-rich medium showed higher expression of KRT3, DSG3 and E-cad, and lower expression of stem cell-related marker ΔNp63 and extracellular matrix proteins laminin and Coll-IV. GJA1 (Gap junction-1, also known as Connexin 43) is a differentiation marker [47, 68] related to the expression of gap junctions, and also showed higher focal expression in cells cultured in CaCl_2_-rich medium. Cells in CnT-PR medium showed aspecific cytoplasmatic and perinuclear GJA1 staining, most probably due to inhibition of GJA1 secretion as cells are not being stimulated to differentiate. Cells in CnT-PR + CaCl_2_ displayed focal intercellular gap junction staining (white arrow). KRT14 expression had shifted from basal cells to suprabasal cells. This might indicate that basal cells had regained their quiescent LESC phenotype, whereas active proliferating suprabasal cells mimicked native transient amplifying cells. INTB4 and INTBA6 expression had shifted from aspecific extracellular expression to membranous co-localization.

*Note: To detect statistical significance for RT-PCR data, independent non-parametric t-testing was performed using the Mann-Whitney U test in Prism 5 (GraphPad software, CA, USA).*
