# Supplementary material for: In Vitro Cultivation of Limbal Epithelial Stem Cells on Surface-Modified Crosslinked Collagen Scaffolds
Source: Stem Cells Int. 2019 Apr 1;2019:7867613. doi: 10.1155/2019/7867613 (PMC6466865; doi:10.1155/2019/7867613)
Supplement: Supplementary 5 — Table S1: antibodies used in immunohistochemical analyses. [file 7867613.f5.docx]

### Table S1: Antibodies used in immunohistochemical analyses

| **Table S1: Antibodies used in immunohistochemical analyses** | | | |
| --- | --- | --- | --- |
| Antibody | Dilution | Specificity | Reference |
| Anti-ΔNp63 | 1:100 | Delta Negative p63 isotype | NBP2-29467, Novus Bio, Littleton, Colorado |
| Anti-Coll-IV | 1:100 | Collagen type IV | Ab6586, Abcam, Cambridge, UK |
| Anti-KRT14 | 1:100 | Cytokeratin 14 | Ab9220, Abcam |
| Anti-Laminin | 1:25 | Laminin | Ab11575, Abcam |
| Anti-KRT3 | 1:100 | Cytokeratin 3 | Ab68260, Abcam |
| Anti-DSG3 | 1:100 | Desmoglein 3 | Ab14416, Abcam |
| Anti-E-cad | 1:100 | E-cadherin | Ab1416, Abcam |
| Anti-INTB4 | 1:50 | Integrin-β4 | Ab110167, Abcam |
| Anti-GJA1 | 1:1000 | Gap Junction-1, known as Connexin 43 (Cx43) | Ab11370, Abcam |
| Cy3 conjugated donkey anti-Rabbit | 1:1000 |  | 711-165-152, Jackson ImmunoResearch (JI), Suffolk, UK |
| Biotin conjugated Donkey anti-Mouse | 1:100 |  | 715-065-151, JI |
| Biotin conjugated Donkey anti-Rabbit | 1:100 |  | 711-065-152, JI |
| Biotin conjugated Donkey anti-Rat | 1:100 |  | 712-065-153, JI |
| Cy3 conjugated Streptavidin | 1:1000 |  | 016-160-084, JI |
| FITC conjugated Streptavidin | 1:100 |  | 016-010-084, JI |
