# Supplementary material for: In Vitro Cultivation of Limbal Epithelial Stem Cells on Surface-Modified Crosslinked Collagen Scaffolds
Source: Stem Cells Int. 2019 Apr 1;2019:7867613. doi: 10.1155/2019/7867613 (PMC6466865; doi:10.1155/2019/7867613)
Supplement: Supplementary 6 — Table S2: relative fold change in gene expression of limbal epithelium cultivated on different carrier materials. [file 7867613.f6.docx]

### Table S2: Relative fold change in gene expression of limbal epithelial cells cultivated on different carrier materials

| **Table S2: Relative fold change in gene expression of limbal epithelium cultivated on different carrier materials** | | | | | | | | | |
| --- | --- | --- | --- | --- | --- | --- | --- | --- | --- |
|  | **HAM** | **RHC I** | **RHC I F-µCP** | **CLP-12 EDC** | **CLP-12** | **CLP-12 F-µCP** | **CLP-12 3D** | **CLP-18** |  |
| **ΔNp63α** | 1.005 ± 0.28 | 1.349 ± 0.41 | 1.058 ± 0.19 | 0.982 ± 0.21 | 1.341 ± 0.26 | 1.122 ± 0.24 | 1.137 ± 0.14 | 1.738 ± 0.26 |  |
| **INTB1** | 0.928 + 0.25 | 0.687 ± 0.08 | 0.528 ± 0.13 | 1.045 ± 0.23 | 1.508 ± 0.09 | 0.856 ± 0.11 | 0.891 ± 0.23 | 1.204 ± 0.29 |  |
| **INTA6** | 0.969 ± 0.18 | 1.206 ± 0.42 | 1.054 ± 0.18 | 2.298 ± 0.74 | 0.892 ± 0.14 | 1.085 ± 0.11 | 1.174 ± 0.11 | 1.579 ± 0.35 |  |
| **KRT3** | 0.107 ± 0.04 | 0.013 ± 0.01 | 0.049 ± 0.03 | 0.137 ± 0.05 | 0.555 ± 0.20 | 0.663 ± 0.27 | 0.730 ± 0.38 | 1.221 ± 0.30 |  |
| **DSG3** | 1.481 ± 0.30 | 2.247 ± 0.93 | 0.904 ± 0.33 | 1.304 ± 0.39 | 1.597 ± 0.73 | 2.234 ± 0.45 | 1.939 ± 0.45 | - 1. 0.10 |  |
